# *Bacillus subtilis* CECT30572 for biological fertilizer stabilization: an alternative to synthetic nitrification inhibitors

**DOI:** 10.3389/fpls.2026.1936312

**Published:** 2026-10-29

**Authors:** Ismael Mazuecos-Aguilera, Pablo Fernández-Carbajo, Ana M. Laureano-Marín, Javier Brañas, Fernando González-Andrés

**Affiliations:** 1 Chemical, Environmental and Bioprocess Engineering Group, Institute for Research and Innovation in Engineering (I4), University of León, León, Spain; 2 Centro de Tecnologías Agroambientales (CTA) Fertiberia - Edificio Centro de Investigación, Tecnología e Innovación de la Universidad de Sevilla (CITIUS) (Centro de Investigación, Tecnología e Innovación) 1, Sevilla, Spain

**Keywords:** *Bacillus subtilis*, biological nitrogen immobilization, microbial plant biostimulant, nitrate assimilation, nitrate leaching, nitrogen use efficiency

## Abstract

**Introduction:**

Modern intensive agriculture faces the critical challenge of reduced nitrogen use efficiency and severe environmental impacts associated with nitrate (NO_3_^−^) leaching. Although chemical nitrification inhibitors such as 3,4-dimethylpyrazole phosphate (DMPP) are widely used to reduce nitrogen losses, there is growing interest in sustainable, bio-based alternatives. This study investigates *Bacillus subtilis* CECT30572, the active biological component of the commercial nitrogen stabilizer NSAFE^®^, as a microbial strategy to reduce nitrate leaching.

**Methods:**

Hybrid whole-genome sequencing was performed to characterize genes associated with nitrogen metabolism. In vitro assays evaluated nitrate depletion, and soil column leaching experiments were conducted under contrasting soil types using different fertilizer formulations.

**Results:**

Hybrid whole-genome sequencing revealed a conserved *nasABCDEF* cluster and regulatory elements (*tnrA, glnR, codY, gltC*) associated with nitrogen assimilation. The genome also encodes the respiratory narGHJI operon for nitrate reduction to nitrite (NO_2_>^−^), while lacking canonical genes for complete denitrification (*nirK/S, norBC, nosZ*), indicating a non-denitrifying nitrogen metabolism. In vitro assays showed substantial nitrate removal, with a net depletion of 455 mg L^−1^ NO_3_^−1^. The nitrate removed from the medium was not recovered predominantly as extracellular or intracellular-associated NH_4_^+^. Although its specific fate cannot be determined from the present measurements, we hypothesize that it could have been assimilated with the bacteria in the form of amino acids. Soil column leaching experiments under contrasting soil types showed that coating or mixed fertilizers (granular NP, granular N, UAN) with CECT30572 significantly reduced cumulative NO_3_^−^ leaching, with reductions of a magnitude similar to those observed with DMPP under several experimental conditions.

**Discussion:**

Overall, these results indicate that the nitrate removal observed for *B. subtilis* CECT30572 under in vitro conditions may contribute to the reduction in nitrate losses observed in soil columns, supporting its potential as a bio-based strategy for reducing nitrate leaching.

## 1 Introduction

Modern intensive agriculture relies heavily on the application of synthetic nitrogen (N) fertilizers to meet global food demands and maximize crop productivity (Erisman et al., 2008; Zhang et al., 2015). However, the nitrogen use efficiency (NUE) of these inputs remains low, often falling below 50% in conventional farming systems (Raun and Johnson, 1999; Lassaletta et al., 2014). This inefficiency is largely due to the rapid biochemical transformations that nitrogen undergoes within the soil matrix, whereby applied ammoniacal or ureic fertilizers are readily converted into highly mobile nitrate (NO_3_^-^) through the sequential nitrification process mediated by autotrophic ammonia- and nitrite-oxidizing microorganisms (Kuypers et al., 2018). Owing to its high solubility and limited retention within the soil matrix, NO_3_^-^ is highly susceptible to transport through the soil profile and subsequent leaching losses (Di and Cameron, 2002; Cameron et al., 2013). Consequently, nitrate leaching constitutes both a substantial agronomic loss and a major environmental concern, contributing to the eutrophication of aquatic ecosystems, contamination of groundwater resources, and indirect greenhouse gas emissions through denitrification pathways (Galloway et al., 2008; Robertson and Vitousek, 2009; Cameron et al., 2013).

To mitigate nitrogen losses and better synchronize nutrient availability with crop nitrogen demand, agriculture has largely relied on chemical nitrogen stabilizers (Cameron et al., 2013; G. P. Robertson and Vitousek, 2009). Among these, synthetic nitrification inhibitors such as 3,4-dimethyl-1H-pyrazole phosphate (DMPP) have been widely adopted. DMPP acts by transiently inhibiting the activity of ammonia monooxygenase (AMO) in autotrophic ammonia-oxidizing bacteria (AOB) and archaea (AOA), thereby slowing the oxidation of NH_4_^+^ to NO_2_^-^ and delaying subsequent nitrate formation (Zerulla et al., 2001; Di and Cameron, 2002). Although synthetic nitrification inhibitors have proven effective in reducing early nitrate production and associated nitrogen losses (Gilsanz et al., 2016), their performance can be strongly influenced by environmental conditions, including soil temperature, moisture, and organic matter content, which determine their persistence and duration of effectiveness (Ruser and Schulz, 2015; Guardia et al., 2018). Furthermore, increasing policy emphasis on reducing dependence on synthetic agrochemical inputs and improving the sustainability of agricultural production systems, as reflected in initiatives such as the European Green Deal, has stimulated interest in the development of bio-based alternatives capable of enhancing fertilizer efficiency while maintaining environmental compatibility (Boix-Fayos and de Vente, 2023).Despite growing interest in the use of beneficial microorganisms to improve nitrogen use efficiency and fertilizer performance, the molecular and physiological mechanisms through which soil microorganisms contribute to nitrogen conservation remain incompletely understood (Beeckman et al., 2024; Koch and Sessitsch, 2024). Although numerous plant growth-promoting rhizobacteria (PGPR) have been described for their beneficial effects on plant nutrition and crop productivity (Olanrewaju et al., 2017; Backer et al., 2018), considerably less attention has been devoted to understanding the genomic basis, physiological mechanisms, and agronomic significance of microbial nitrate assimilation as a strategy for nitrogen conservation (Koch and Sessitsch, 2024; Wang and Song, 2024).

In this context, plant growth-promoting rhizobacteria (PGPR) have emerged as promising tools for the development of bio-based strategies aimed at improving NUE and mitigating nitrogen losses from agricultural systems (Backer et al., 2018). Soil microorganisms can influence nitrogen cycling through multiple mechanisms, ranging from limiting the availability of ammonium for nitrifiers through competition to the temporary immobilization of inorganic nitrogen within microbial biomass. Such biological processes have the potential to reduce the immediate susceptibility of mineral nitrogen to loss while preserving its availability within the soil–plant system (Kuzyakov and Xu, 2013; Schimel and Bennett, 2004). Among soil-associated PGPR, the genus *Bacillus* has attracted particular interest owing to its remarkable metabolic versatility, resilience to environmental stresses, and ability to form highly resistant endospores, traits that facilitate industrial-scale production, formulation, and long-term viability following fertilizer coating (Borriss, 2011). Although *Bacillus* spp. are widely recognized for their roles in phosphate solubilization, phytohormone production, induced systemic resistance, and biological control of plant pathogens (Poveda & González-Andrés, 2021), their contribution to nitrogen conservation through assimilatory nitrate metabolism has received comparatively little attention.

The present study focuses on *Bacillus subtilis* CECT30572, the active biological component of the commercial fertilizer stabilizer NSAFE^®^ (Fertiberia). Although previous field observations confirm that this strain influences nitrate availability in the rhizosphere, thereby reducing nitrogen losses (González-Andrés et al., 2025), the molecular basis, physiological mechanisms, and agronomic relevance of this phenotype remain unresolved. In particular, its genomic determinants and functional capacity for nitrate cycling have not been experimentally validated.

Accordingly, this study aimed to elucidate the molecular basis of nitrate assimilation in *Bacillus subtilis* CECT30572 and its impact on nitrate leaching under agronomically relevant conditions. Specifically, the objectives were to: (i) resolve the chromosomal architecture, syntenic conservation, and transcriptional regulatory network associated with nitrogen assimilation through hybrid whole-genome sequencing; (ii) functionally characterize the kinetics of nitrate assimilation *in vitro* by monitoring nitrate depletion and the dynamics of nitrogen metabolic intermediates under nitrate as the sole nitrogen source; and (iii) to evaluate whether this biological mechanism translates into reduced nitrate leaching under soil leaching columns with contrasting soil types by comparing CECT30572 with the synthetic nitrification inhibitor DMPP across different fertilizer formulations (granular NP and N, and liquid UAN).

## 2 Materials and methods

### 2.1 Whole-genome sequencing and bioinformatics

The whole genome of *Bacillus subtilis* CECT30572 was sequenced using a hybrid next-generation sequencing (NGS) approach combining Oxford Nanopore (long reads) and Illumina (short reads) platforms, performed by MicrobesNG (Birmingham, UK). Subsequent *de novo* genome assembly and functional annotation were performed using the open-source bioinformatics platform Galaxy (Abueg et al., 2024). Quality control of the raw sequencing data was assessed using FastQC (v0.74+galaxy1) (Wingett and Andrews, 2018), and low-quality bases and reads shorter than 50 bp were trimmed using Fastp (v1.1.0+galaxy0) with a Phred score threshold of Q20. Hybrid *de novo* assembly was carried out using Unicycler (v0.5.1+galaxy) (Wick et al., 2017). Assembly statistics were assessed using QUAST (v5.3.0+galaxy1) (Gurevich et al., 2013; Mikheenko et al., 2018). Genome completeness was assessed using BUSCO (v6.1.0) with the *bacteria_odb12* lineage dataset (Manni et al., 2021), while genome completeness and contamination were independently estimated using CheckM2 (v1.1.0) (Chklovski et al., 2023). Genome annotation was performed using Prokka (v1.14.6+galaxy1) (Seemann, 2014), and the presence of plasmid sequences was screened using PlasmidFinder (v2.1.6+galaxy1) (Carattoli and Hasman, 2020). The resulting hybrid assembly consisted of a single circularized contig. Terminal overlaps were resolved during the assembly process, and no additional post-assembly polishing was performed.

Genome-based taxonomic assignment of CECT30572 was further assessed using average nucleotide identity (ANI), digital DNA–DNA hybridization (dDDH), and phylogenomic analysis. ANI was calculated using FastANI by comparing the CECT30572 genome against the genome of *B. subtilis* ATCC 6051. Genome-wide dDDH values and genome-based phylogenomic placement were obtained using the Type (Strain) Genome Server (TYGS), which applies the Genome BLAST Distance Phylogeny (GBDP) approach. The resulting phylogenomic tree was used to assess the placement of CECT30572 relative to closely related *Bacillus* genomes.

The complete genome sequence has been deposited in the NCBI database under BioSample accession SAMN61482408 and BioProject accession PRJNA1492302, with the genome accession JCAMQZ000000000.

### 2.2 Comparative synteny

Synteny analyses were conducted to evaluate the genomic architecture of genes involved in assimilatory nitrogen metabolism across representative *Bacillus* species. In addition to the target strain *Bacillus subtilis CECT30572*, several representative strains were analyzed, with *Bacillus subtilis* subsp. *subtilis* str. 168 (NC_000964.3) serving as the primary reference due to its well-characterized nitrogen regulatory networks (Borriss et al., 2018). The wild-type *B. subtilis* subsp. *subtilis* str. NCIB 3610 (CP034484.1) was included to account for potential laboratory domestication effects (Branda et al., 2001). NCIB 3610 is an alternative designation of the *B. subtilis* type strain ATCC 6051 and was used here specifically as the non-domesticated reference for the synteny analysis; whereas ATCC 6051 was used as the representative type-strain reference for the ANI, dDDH, and TYGS phylogenomic analyses. *B. subtilis* subsp. *spizizenii* (CP002905.1) was used to determine conservation within the *subtilis* complex (Earl et al., 2012). Additionally, three agriculturally relevant plant-associated strains were incorporated: *Bacillus velezensis* NRRL B-41580 (NC_009725.2) (Lv et al., 2025)*, Bacillus amyloliquefaciens* DSM7 (NC_014551.1) (Rückert et al., 2011), and the emerging taxon *Bacillus cabrialesii* TSO2 (CP096889.1) (de los Santos Villalobos et al., 2019). Lastly, *Priestia megaterium* str. NBRC 15308 (CP035094.1) was selected as a phylogenetically distant representative to assess the broader conservation of these metabolic pathways.

To identify and visualize gene cluster conservation, pairwise protein sequence alignments of the target genomic regions were performed using clinker (v0.1.3) through the CAGECAT web platform (Gilchrist and Chooi, 2021). Comparative synteny blocks, gene-order collinearity, and homology groups were visualized using clinker. Protein sequence identity values obtained from the pairwise alignments were used to generate a comparative identity matrix among the analyzed genomes. Homology groups were determined using default parameters.

### 2.3 Bacterial strain and culture maintenance

The strain CECT30572, originally isolated from agricultural soils, is the active microbial component of the commercial product NSAFE^®^ (Fertiberia), which also contains additional formulation ingredients for agricultural application. Stock cultures were maintained at −80 °C in 25% (v/v) glycerol. Glycerol scrapes were streaked on tryptic soy agar (TSA, Sigma-Aldrich) plates and incubated at 28 °C overnight. Single colonies were then used to inoculate 100 mL of tryptic soy broth (TSB, Sigma-Aldrich) and incubated overnight at 28 °C with orbital shaking at 115 rpm to obtain pre-inocula.

### 2.4 Nitrate minimal medium and growth conditions

Assimilatory nitrate (NO_3_^-^) reduction by CECT30572 was studied in triplicate using a NO_3_^-^ minimal medium (NMM), in which NO_3_^-^ was provided as the sole nitrogen source. The medium contained (per liter): 1 g KNO_3_, 0.6 g KH_2_PO_4_, 0.4 g MgSO_4_·7H_2_O, 4 g glucose, 8 g mannitol, 8 g sodium pyruvate, 100 µg thiamine, 25 µg biotin, 0.2 g FeCl_3_, and 0.4 g CaCl_2_ adapted from Bergersen (1961). The pH was adjusted to 6.8 prior to sterilization.

To initiate the NMM cultures, 1 mL of a previously prepared pre-inoculum was centrifuged at 4,000 × *g* for 20 min. The resulting bacterial pellet was washed twice with 0.9% (w/v) NaCl and subsequently used as the inoculum for NMM. The culture was maintained at 28 °C with orbital shaking at 115 rpm.

Samples were collected from each replicate at designated time intervals over a 203 h period (0–203 h). For the determination of viable bacterial cell density, colony-forming units (CFU mL^-^¹) were quantified via serial dilution plating. In parallel, aliquots were harvested for chemical tracking: one fraction was immediately filtered through 0.45 µm pore-size filters and stored at −20 °C for subsequent analysis of extracellular NO_3_^-^, nitrite (NO_2_^-^), and ammonium (NH_4_^+^) concentrations. For the determination of total NH_4_^+^, including both cellular and extracellular fractions, separate aliquots were stored unfiltered at −20 °C. Prior to chemical analysis, these unfiltered samples were subjected to sonication to achieve complete cell lysis and were subsequently clarified through 0.45 µm pore-size filters.

### 2.5 Soil leaching column experiment

#### 2.5.1 Spore production and fertilizer coating

For spore production intended for fertilizer coating, 1 mL of the pre-inoculum was used to establish cultures of CECT30572 in a molasses-based medium (2.3% v/v). Cultures were incubated for 48 h at 28 °C and 115 rpm. Spore concentration was determined by serial dilution plating after heat treatment at 80 °C for 20 min. The molasses medium promotes sporulation of the bacterial strain enhancing survival following application to the fertilizer surface. The suitability of molasses for fermentation and sporulation processes has been previously demonstrated by (Pastor-Bueis et al., 2017).

Granular fertilizers (NP and N) were mechanically coated at a dose of 300 µL of the mature molasses spore culture per 100 g of fertilizer and subsequently air-dried for 24 h prior to soil application. For the liquid fertilizer (UAN), an equivalent biological dose was calculated based on its density and thoroughly pre-mixed into the liquid matrix immediately before application. For DMPP treatments, the commercial DMPP formulation containing 17.4% active DMPP was applied at rates of 5.52 mg formulation g^-^¹ fertilizer for granular NP, 13.79 mg formulation g^-^¹ fertilizer for granular N, and 11.03 mg formulation g^-^¹ fertilizer for UAN. These rates corresponded to 0.8% of the nitrifiable N present in each fertilizer, expressed as active DMPP.

#### 2.5.2 Viable bacterial counts in fertilizer formulations during storage

The persistence of viable bacteria associated with the CECT30572 inoculation in the different fertilizer formulations was evaluated at 0, 6, and 12 months during storage. A separate mature molasses spore culture, with a concentration of 7.1 × 10¹^0^ CFU mL^-^¹, was used for fertilizer inoculation. Granular fertilizers (NP and N) were coated with 300 µL of this bacterial culture per 100 g of fertilizer and subsequently air-dried for 24 h. For the liquid fertilizer (UAN), an equivalent biological dose was calculated based on its density and thoroughly pre-mixed into the liquid matrix prior to storage. For each fertilizer formulation and sampling time, three independent inoculated fertilizer samples and three corresponding non-inoculated control samples were analyzed (n = 3 independent storage units per treatment and sampling time). Each sample was stored independently in a separate hermetically sealed container throughout the storage period, and these independently stored samples constituted the experimental units. Fertilizers were stored in a dry, dark location at room temperature. Ambient temperature was not actively controlled, and relative humidity was not monitored during the storage period.

For granular fertilizers, 15 g of fertilizer was suspended in 15 mL of sterile distilled water and thoroughly homogenized. Ten-fold serial dilutions of the resulting suspension were then prepared in sterile saline solution, and aliquots were spread-plated on tryptic soy agar (TSA). For liquid UAN, samples were directly subjected to ten-fold serial dilution in sterile saline solution and plated on TSA. The same procedures were applied to inoculated and non-inoculated fertilizer samples. For each independent fertilizer sample, three independent dilution/recount measurements were performed, with three TSA plates analyzed for each measurement. The three independent storage units and their three corresponding recount measurements per unit resulted in nine numerical observations per fertilizer formulation and sampling time. These nine observations were used to calculate the descriptive mean and standard deviation; no inferential statistical analysis was performed for the storage experiment. Plates were incubated at 28 °C for 24 h, after which colony-forming units (CFU) were determined. Results were expressed as CFU g^-^¹ for granular fertilizers and CFU mL^-^¹ for liquid UAN. For each fertilizer formulation and sampling time, the viable bacterial count attributable to the inoculation was calculated by subtracting the count obtained for the corresponding non-inoculated fertilizer control from the count obtained for the inoculated formulation.

#### 2.5.3 Soil characteristics and column design

To evaluate the potential of CECT30572 to mitigate NO3- leaching from different nitrogen sources, two contrasting soils were tested. The first soil (S1) was collected from Montemor-o-Velho, Coimbra, Portugal (collection date: 20 November 2024; coordinates: 40° 11’ 40,78” N, 8° 35’ 07,25” W) and the second soil (S2) was collected from Cacabelos, León, Spain (collection date: 14 November 2024; coordinates: 42° 36′ 25.9″ N, 6° 45′ 18.1″ W). Both soils were air-dried, sieved through a 2 mm mesh and mixed with commercial silica sand (0.5 mm grain size) at a 6:1 (v/v) soil-to-sand ratio. The resulting mixture was subsequently combined with vermiculite n°. 3 at a 3:1 (v/v) mixture-to-vermiculite ratio to obtain the final substrate. The main physicochemical properties of the substrates are shown in **Table 1**.

**Table 1:** Physicochemical analysis of the substrates used in the leaching column experiment.

Soil analysis		S1 (Portugal)	S2 (Spain)
Texture	Sand (%)	56	50
	Silt (%)	34	32
	Clay (%)	10	18
	Textural class (USDA)	SANDY LOAM	LOAM
Field capacity (%)		37.77	35.14
Saturation (%)		45	43
pH (water extract)		5.47	5.12
Carbonates (%CaCO_3_)		trace	trace
Organic matter (%)		4.51	2.33
Total nitrogen (%)		0.14	0.09
C/N ratio		18.80	15.90
Phosphorus (Olsen) (ppm)		32.36	13,21
Cations	Calcium (cmol (+) kg^‐1^)	5.11	4.37
	Magnesium (cmol (+) kg^‐1^)	1.85	3.54
	Potassium (cmol (+) kg^‐1^)	0.50	0.28
	Sodium (cmol (+) kg^‐1^)	0.08	0.05
Ca/Mg ratio		2.76	1.23
K/Mg ratio		0.27	0.08
C.E.C. (cmol (+) kg^‐1^) (cation Exchange capacity)		7.72	8.00
Microelements	Manganese (ppm)	12.66	10.48
	Iron (ppm)	112.85	48.63
	Copper (ppm)	1.17	7.81
	Zinc (ppm)	2.60	10.09
Boron (ppm)		0.33	0.38
Electrical conductivity (dS/m)		0.03	0.21

The leaching assay was conducted using lysimeter-type columns constructed from PVC tubes (250 mm in diameter, 250 mm in height), adapted from the design by (Sartain et al., 2003). The bottom of each column was sealed with a perforated (5 mm) PVC cap and lined internally with a double 1 mm nylon mesh to prevent substrate loss. Each column was uniformly packed with 3.5 kg of the corresponding substrate mixture.

#### 2.5.4 Experimental treatments and fertilization doses

Three distinct fertilizer prototypes were evaluated: (i) granular NP (NP), containing 20% N (12% ammoniacal; 8% nitric); (ii) granular N (N), containing 30% N (5% ammoniacal; 25% ureic); and (iii) liquid ammonium nitrate-urea solution UAN (UAN), containing 32% N (8% ammoniacal; 16% ureic; 8% nitric).

For each nitrogen matrix, four stabilization strategies were compared: an uncoated positive control (C+), a chemical control stabilized with the commercial nitrification inhibitor 3,4-dimethyl-1H-pyrazole (DMPP), a biological treatment coated with CECT30572 (CECT30572), and an unfertilized negative control (C−). A detailed matrix of all experimental treatments is presented in **Table 2**.

**Table 2:** Treatment abbreviations, descriptions, and fertilizers evaluated in each substrate in the leaching column experiment.

Substrate	Fertilizer	Treatments	N application	Stabilizer dose
S1	Granular NP	C-	Unfertilized	–
		Granular NP/C+	114.26 mg N kg^-^¹	–
		Granular NP/DMPP	114.26 mg N kg^-^¹	11.04 mg commercial DMPP formulation column^-^¹ (5.52 mg formulation g^-^¹ fertilizer)
		Granular NP/CECT30572	114.26 mg N kg^-^¹	4.06 × 10^6^ CFU column^-^¹ (2.03 × 10^6^ CFU g^-^¹ fertilizer)
	Granular N	C-	Unfertilized	–
		Granular N/C+	171.43 mg N kg^-^¹	–
		Granular N/DMPP	171.43 mg N kg^-^¹	27.58 mg commercial DMPP formulation column^-^¹ (13.79 mg formulation g^-^¹ fertilizer)
		Granular N/CECT30572	171.43 mg N kg^-^¹	4.06 × 10^6^ CFU column^-^¹ (2.03 × 10^6^ CFU g^-^¹ fertilizer)
S2	Granular NP	C-	Unfertilized	–
		Granular NP/C+	114.26 mg N kg^-^¹	–
		Granular NP/DMPP	114.26 mg N kg^-^¹	11.04 mg commercial DMPP formulation column^-^¹ (5.52 mg formulation g^-^¹ fertilizer)
		Granular NP/CECT30572	114.26 mg N kg^-^¹	2.34 × 10^6^ CFU column^-^¹ (1.17 × 10^6^ CFU g^-^¹ fertilizer)
	UAN	C-	Unfertilized	–
		UAN/C+	182.86 mg N kg^-^¹	–
		UAN/DMPP	182.86 mg N kg^-^¹	22.06 mg commercial DMPP formulation column^-^¹ (11.03 mg formulation g^-^¹ fertilizer)
		UAN/CECT30572	182.86 mg N kg^-^¹	2.34 × 10^6^ CFU column^-^¹ (1.17 × 10^6^ CFU g^-^¹ fertilizer)

*DMPP was applied at 0.8% of the nitrifiable N present in each fertilizer, expressed as active DMPP. The corresponding doses of the commercial DMPP formulation (17.4% DMPP) were calculated based on its active ingredient concentration. CECT30572 was applied at 300 µL of bacterial suspension per 100 g of fertilizer. The bacterial suspension contained 6.77 × 10^8^ CFU mL^-^¹ for S1 treatments and 3.90 × 10^8^ CFU mL^-^¹ for S2 treatments, corresponding to 4.06 × 10^6^ and 2.34 × 10^6^ CFU column^-^¹, respectively, based on the 2 g of fertilizer applied per column.

Four independent experimental replicates (n = 4 columns) were established per treatment. Each soil column constituted one independent experimental unit. Each fertilized column received a fixed dose of 2 g of granular fertilizer (or an equivalent volume of 1538 µL for the liquid UAN, based on its density of 1.3 g mL^-1^), which established individual nitrogen inputs of 114.26 mg N kg^-1^ of substrate for granular NP, 171.43 mg N kg^-1^for granular N, and 182.86 mg N kg^-1^for UAN. All fertilizers were carefully incorporated into the top 5–7 cm of the column substrate layer to simulate field incorporation. For the chemical stabilization treatments, the commercial DMPP formulation containing 17.4% active DMPP was applied at a dose corresponding to 0.8% of the nitrifiable N present in each fertilizer, expressed as active DMPP. Nitrifiable N was calculated as the sum of ammoniacal and ureic N, excluding nitrate-N. This resulted in application rates of 5.52 mg DMPP formulation g^-^¹ fertilizer for granular NP, 13.79 mg DMPP formulation g^-^¹ fertilizer for granular N, and 11.03 mg DMPP formulation g^-^¹ fertilizer for UAN. Based on the 2 g of fertilizer applied per column, these rates corresponded to 11.04 mg DMPP formulation column^-^¹ for granular NP, 27.58 mg DMPP formulation column^-^¹ for granular N, and 22.06 mg DMPP formulation column^-^¹ for UAN. For the biological stabilization treatments, CECT30572 was applied at a rate of 300 µL of bacterial suspension per 100 g of fertilizer. The bacterial suspension contained 6.77 × 10^8^ CFU mL^-^¹ for the S1 treatments and 3.90 × 10^8^ CFU mL^-^¹ for the S2 treatments. Considering the 2 g of fertilizer applied per column, these application rates corresponded to 4.06 × 10^6^ CFU CECT30572 column^-^¹ for both granular NP and granular N in S1, and 2.34 × 10^6^ CFU column^-^¹ for granular NP and UAN in S2. The corresponding application rates are summarized in **Table 2**.

#### 2.5.5 Incubation conditions and leaching events

The experimental columns were arranged inside a phytotron maintained at a constant temperature of 20 °C in complete darkness. The top of each column was covered with filter paper to minimize evaporative water loss while allowing unrestricted gas exchange. Immediately following treatment application, the substrates were progressively moistened with distilled water up to field capacity, ensuring full wetting of the fertilized top layer while strictly avoiding any early leachate formation.

After an initial static incubation period of one week, five consecutive weekly leaching events were carried out. At each event, distilled water was applied in a volume equivalent to the difference between the saturation capacity and field capacity of each specific substrate. Leachates were allowed to drain freely for 24 h before collection. Total leachate volumes were recorded, and after thorough homogenization, representative aliquots were sampled and stored at −20 °C for subsequent NO_3_^-^ analysis.

### 2.6 Nitrate, nitrite, and ammonium analysis in NMM culture and column leachates

Nitrate (NO_3_^-^) was quantified in both NMM culture samples and column leachates. Nitrite (NO_2_^-^) and ammonium (NH_4_^+^) were quantified only in NMM culture samples.

Nitrate and nitrite analyses were performed by ion chromatography using a Metrohm 881 Compact IC Pro system equipped with a Metrosep A Supp 7 column and a high-resolution conductivity detector with inline chemical suppression. The mobile phase consisted of 3.6 mM sodium carbonate. Calibration was carried out using a commercial mixed anion standard solution (nitrate and nitrite; 1,000 mg L^-^¹, E3A7.1k.W.L1, CPA Chem). A six-point calibration curve (0.1, 0.5, 1, 5, 10, and 20 mg L^-^¹) was prepared by serial dilution of the stock standard, and concentrations were calculated based on peak area responses. Prior to analysis, samples were diluted 10- and 100-fold in deionized water to fit within the calibration range.

Ammonium (NH_4_^+^) was determined spectrophotometrically using a Pharo 300 spectrophotometer (Merck) and Spectroquant^®^ Ammonium Cell Test kits (Supelco), following the manufacturer’s instructions. Samples were diluted in deionized water prior to analysis (5-fold dilution for most samples and 10-fold dilution for samples collected at 173 h and 197 h) to ensure compatibility with the assay range.

### 2.7 Statistical analyses

Data normality and homoscedasticity were evaluated using the Shapiro-Wilk and Levene tests, respectively. To account for the repeated measurements obtained from the same soil columns across successive leaching events, linear mixed-effects models were fitted, including treatment, leaching event, and their interaction as fixed effects, with soil column as the subject and an autoregressive AR(1) covariance structure for repeated measurements. Thus, the soil column was the independent experimental unit (n = 4 per treatment), whereas the five leaching events represented repeated observations within each experimental unit and were not considered independent replicates. When a significant treatment × leaching event interaction was detected, treatment differences at individual leaching events were further examined using one-way analysis of variance (ANOVA), followed by Tukey’s *post-hoc* test for multiple comparisons.

For cumulative nitrate mass, only the final cumulative value at the last leaching event was considered as the endpoint representing total nitrate loss over the experimental period. These final cumulative values were analyzed using one-way ANOVA, followed by Tukey’s *post-hoc* test for multiple comparisons. For supplementary analysis, cumulative nitrate-N losses at the final leaching event were also expressed as a percentage of the total nitrogen applied with the corresponding fertilizer treatment. Nitrate-N losses were calculated from measured NO_3_^-^ concentrations using the molecular mass ratio of N to NO_3_^-^ (14/62). All statistical analyses were performed using IBM SPSS Statistics software (v29.0; IBM Corp., Armonk, NY, USA).

## 3 Results

### 3.1 Genome sequencing, assembly, and general features

Hybrid whole-genome sequencing of *Bacillus subtilis* CECT30572 yielded 572,865 Oxford Nanopore reads totaling 1.658 Gb, with a read N50 of 12,288 bp and a mean read quality of Q15.9. These data corresponded to an estimated long-read sequencing depth of 404.8× relative to the assembled genome. Hybrid assembly using Unicycler v0.5.1 yielded a single circularized contig of 4,095,377 bp, with an average G+C content of 43.84%. The assembly showed maximum contiguity, with N50 and N75 values of 4,095,377 bp and L50 and L75 values of 1, and contained no ambiguous bases (Ns). A total of 565,639 Illumina paired-end reads were obtained after quality trimming, corresponding to 280,318,658 bases and an estimated theoretical sequencing depth of 68.4× relative to the assembled genome (**Table 3**).

**Table 3:** Genome assembly, sequencing, annotation and quality metrics of *Bacillus subtilis* CECT30572.

Category	Metric	Result
Assembly	Genome size (bp)	4,095,377
	Number of contigs	1
	Largest contig (bp)	4,095,377
	N50 (bp)	4,095,377
	N75 (bp)	4,095,377
	L50	1
	L75	1
	GC content (%)	43.84
	Ambiguous bases (Ns per 100 kbp)	0.00
Oxford Nanopore sequencing	Number of reads	572,865
	Total bases (Gb)	1.658
	Mean read length (bp)	2,893.6
	Median read length (bp)	553
	Read N50 (bp)	12,288
	Maximum read length (bp)	140,088
	Mean read quality (Q-score)	15.9
	Reads ≥Q20 (%)	31.9
	Estimated sequencing depth (×)	404.8
Illumina sequencing	Paired-end reads (pairs)	565,639
	Bases after trimming (bp)	280,318,658
	Theoretical sequencing depth (×)	68.4
Annotation	Protein-coding sequences (CDS)	4,044
	tRNA genes	86
	rRNA genes	30
	tmRNA genes	1
	Hypothetical proteins	1,198 (29.6%)
Genome completeness	BUSCO complete (%)	99.1
	BUSCO complete, single-copy (%)	96.6
	BUSCO complete, duplicated (%)	2.6
	BUSCO fragmented (%)	0.0
	BUSCO missing (%)	0.9
Genome quality	CheckM2 completeness (%)	100.0
	CheckM2 contamination (%)	0.02

BUSCO, Benchmarking Universal Single-Copy Orthologs; CDS, coding sequences. Estimated ONT sequencing depth was calculated from the total number of Oxford Nanopore bases relative to the assembled genome size. Theoretical Illumina sequencing depth was calculated from the total number of Illumina bases after trimming relative to the assembled genome size.

Functional annotation identified 4,044 coding sequences (CDSs), 86 tRNA genes, 30 rRNA genes, and one tmRNA gene. No plasmid sequences were detected during genomic screening with PlasmidFinder (**Table 3**).

Genome quality assessment indicated a highly complete genome with negligible contamination. BUSCO analysis identified 115 complete BUSCOs out of 116 conserved bacterial genes, corresponding to 99.1% completeness, including 96.6% single-copy and 2.6% duplicated BUSCOs, while no fragmented BUSCOs were detected and only 0.9% were missing. CheckM2 independently estimated 100.0% genome completeness and 0.02% contamination. Together, these metrics support the high completeness and overall quality of the CECT30572 genome assembly (**Table 3**).

### 3.2 Genome-based taxonomic assignment

Genome-based analyses further supported the taxonomic assignment of CECT30572 as *Bacillus subtilis*. FastANI analysis showed 98.67% average nucleotide identity (ANI) between the CECT30572 genome and the genome of *B. subtilis* type strain ATCC 6051. Genome-to-genome comparisons performed using the TYGS platform yielded dDDH values of 94.1%, 90.0%, and 95.5% for the comparison with *B. subtilis* type strain ATCC 6051, depending on the formula applied. (**Supplementary Table S1**).

The TYGS whole-genome sequence-based phylogenomic tree further placed CECT30572 within the *B. subtilis* clade, clustering with the type strain ATCC 6051 (**Supplementary Figure S1**). Together, the high ANI and dDDH values and the phylogenomic placement consistently support the identification of CECT30572 as *B. subtilis*.

### 3.3 Identification and genomic organization of the nitrogen metabolism cluster

Genomic analysis of CECT30572 confirmed the presence of the *nasABCDEF* gene cluster. This locus comprises the *nasA* gene (encoding a putative nitrate/nitrite transporter) transcribed divergently from the *nasBCDEF* operon, which includes *nasBC* (nitrate reductase subunits), *nasDE* (nitrite reductase subunits), and *nasF* (uroporphyrin-III C-methyltransferase).

Additionally, key regulatory genes driving nitrogen homeostasis were identified in the chromosome, including the global transcriptional regulators *tnrA*, *codY*, and *glnR*. The LysR-family activator *gltC* was also detected upstream of the *gltAB* glutamate synthase operon. Finally, the transmembrane ammonium transport system was found to be encoded by the *nrgAB* operon (*amtB* and *glnK*).

In addition to this primary assimilatory apparatus, genomic screening revealed that the strain lacks the complete genomic machinery required for respiratory or dissimilatory reduction of NO_3_^-^ to NH_4_^+^. It possesses specific components associated with respiratory nitrate reduction from NO_3_^-^ to NO_2_^-^ under anaerobic or microaerophilic conditions. Specifically, the chromosome harbors the *narGHIJ* operon (encoding the membrane-bound respiratory nitrate reductase complex) and its associated transporter *narK*, which operate under the regulatory control of the oxygen-responsive global systems *fnr* and *resDE*. However, it lacks the genomic capacity to reduce NO_2_^-^ to NH_4_^+^. Moreover, among the enzymatic machinery typically involved in denitrification, the genome of CECT30572 completely lacks the downstream genes required to further reduce nitrite to gaseous forms (NO, N_2_O, and N_2_). This is evidenced by the total absence of nitrite reductases (*nirK* or *nirS*), nitric oxide reductases (*norBC* or *norZ*), and nitrous oxide reductases (*nosZ*).

### 3.4 Comparative synteny of the nitrogen metabolism loci

Comparative genomic analysis of the chromosomal regions associated with nitrate/nitrite assimilation, nitrogen regulation, and ammonium transport revealed a high degree of structural conservation in CECT30572 compared to reference strains (**Figure 1**). The genomic region containing the *nasA* transporter and the *nasBC* and *nasDEF* operons exhibited strong syntenic conservation and gene-order collinearity with homologous loci in the model strains *B. subtilis* str. 168 and *B. subtilis* str. NCIB 3610 (**Figure 1**), with protein sequence identity values ranging from 97% to 100% (**Supplementary Table S2**). This structural conservation was also observed across other members of the *B. subtilis* group, although the degree of protein sequence conservation varied among genes and species. In *B. cabrialesii*, protein sequence identities for the six *nas* genes ranged from 90% to 96%, whereas lower identities were observed for some genes in *B. subtilis* subsp. *spizizenii* and *B. velezensis*, particularly for *nasF*, *nasB*, and *nasA* (**Supplementary Table S2**).

**Figure 1:**
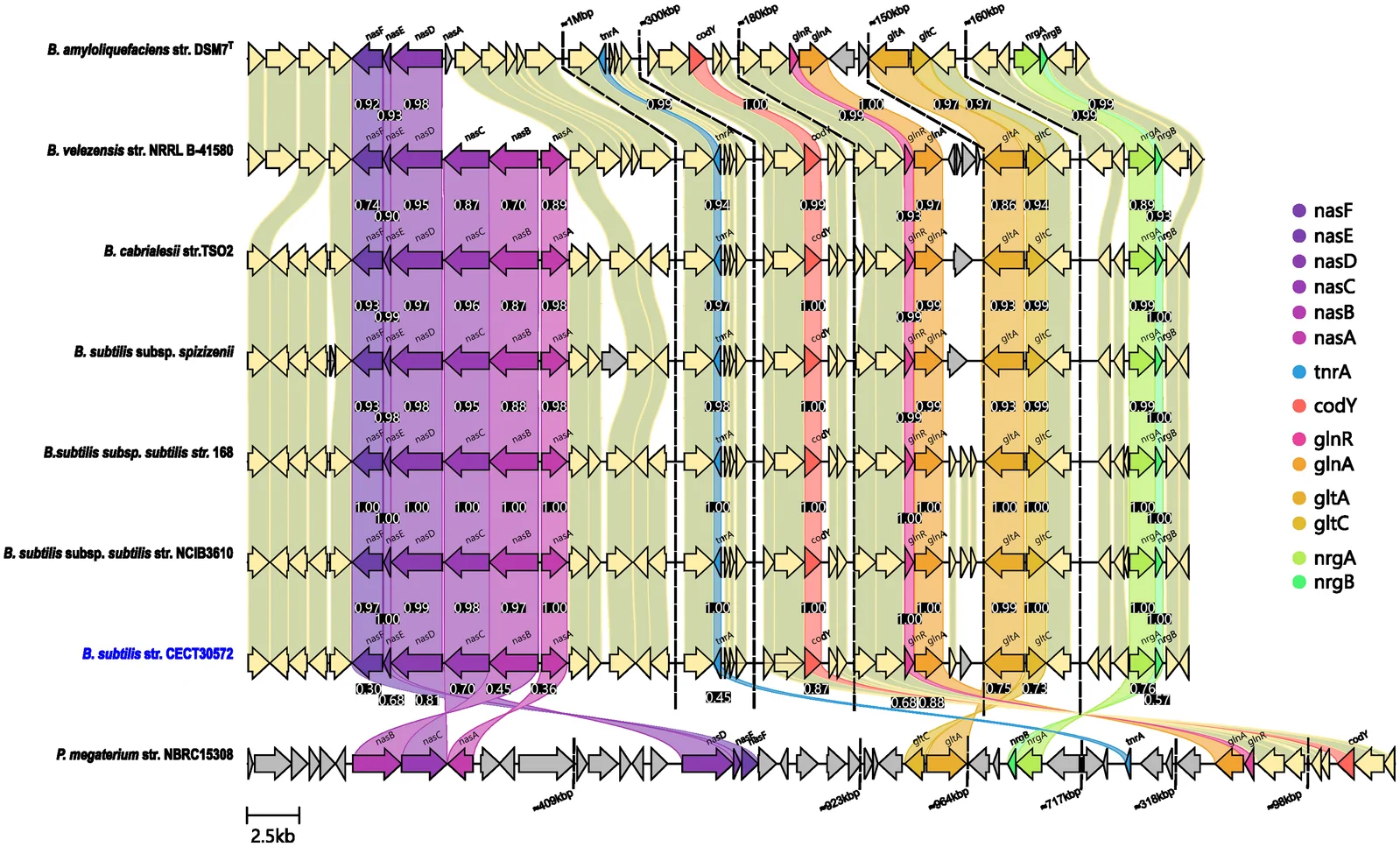
Comparative synteny of genomic regions involved in assimilatory nitrogen metabolism, regulation, and transport between *Bacillus subtilis* CECT30572 (strain name highlighted in blue) and seven closely related *Bacillus* and *Priestia* strains. Gene clusters associated with nitrate and nitrite assimilation (*nasABCDEF*), ammonium transport and assimilation (*nrgAB*, *glnA*), and regulatory control of nitrogen metabolism (*tnrA*, *glnR*, *codY*, *gltC*) were identified in each genome and visualized using clinker via CAGECAT (Gilchrist and Chooi, 2021). Arrows represent predicted coding DNA sequences (CDS), with distinct colors indicating homologous gene groups. Shaded links connect homologous loci, with numbers indicating pairwise sequence similarity values (0–1). Grey-colored CDS indicate the absence of detectable homology, whereas pale yellow represents homologous CDS adjacent to the genomic region of interest but not directly involved in assimilatory nitrogen metabolism.

Beyond the core assimilatory cluster, the global regulators *tnrA* and *codY*, the transcriptional regulators *glnR* and *gltC*, and the *nrgAB* ammonium transport system also exhibited conservation of genomic position and sequence across the analyzed *Bacillus* genomes.

Conversely, distinct genomic variations were observed when comparing flanking regions or more phylogenetically distant taxa. While minor lineage-specific genomic rearrangements were noted in the flanking regions within the *subtilis* group, significantly lower syntenic conservation was observed in *Priestia megaterium*. In this outgroup species, genes associated with nitrogen metabolism remained detectable, but their genomic context and gene order showed extensive divergence. Notably, no corresponding *nasBC* homologs were detected in the analyzed genomic region of *Bacillus amyloliquefaciens* DSM 7 under the alignment conditions used (**Figure 1**).

### 3.5 Growth kinetics and nitrogen source depletion

To validate the functional architecture of the identified operons, the strain CECT30572 was evaluated in minimal medium containing nitrate (NO_3_^-^) as the unique nitrogen source. The strain displayed a classic bacterial growth curve divided into four distinct phases (**Figure 2**). After a 24 h lag phase, exponential replication drove cell density to a maximum of 1.2 x 10^8^ CFU mL^-^¹at 53 h. The stationary phase sustained high cell viability (1.2 x 10^8^–6.6 x 10^7^ CFU mL^-^¹) until 101 h, after which a progressive death phase occurred, reducing cell counts to 1.5 x 10^4^ CFU mL^-^¹ by 203 h.

**Figure 2:**
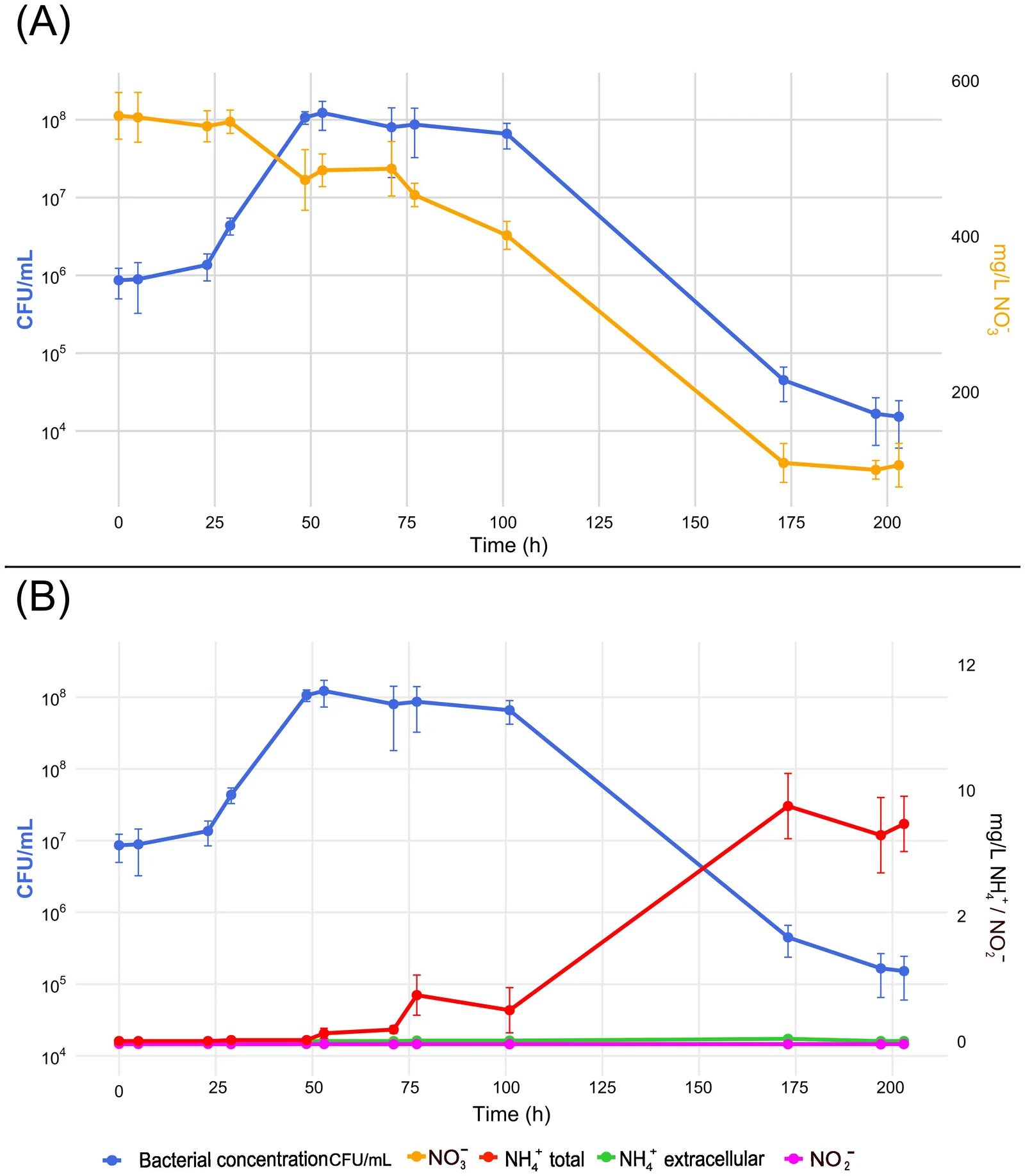
*Bacillus subtilis* CECT30572 growth curve in nitrate minimal medium (NMM) and temporal changes in nitrogen species during culture. **(A)** Bacterial concentration (CFU mL^-^¹, blue line) and nitrate concentration (NO_3_^-^, mg L^-^¹, orange line) over time. **(B)** Bacterial concentration (CFU mL^-^¹, blue line), ammonium concentration on extracellular medium (green line; mg L^-^¹ NH_4_^+^) and total ammonium concentration (extracellular and intracellular; red line; mg L^-^¹ NH_4_^+^) and nitrite concentration (NO_2_^-^, mg L^-1^, pink line) over time. Bacterial abundance is shown on a logarithmic scale, whereas nitrogen species concentrations are shown on linear scales. Time is expressed in hours (h).

NO_3_^-^ consumption profiles strongly correlated with these growth phases, operating in a high concentration range (**Figure 2a**). Initial NO_3_^-^ levels (555 mg L^-1^) decreased steadily during active biomass accumulation, undergoing a continuous depletion through the stationary and death phases to reach a final baseline of 100 mg L^-1^ at 197 h, representing a net consumption of approximately 455 mg L^-1^of NO_3_^-^. Notably, spectrophotometric tracking revealed that nitrite (NO_2_^-^) levels remained strictly undetected in the culture medium throughout the entire duration of the assay.

Parallel tracking of ammonium (NH_4_^+^) generation revealed that free extracellular NH_4_^+^ remained low over the course of 203 h. Meanwhile, total NH_4_^+^ (consisting almost exclusively of the intracellular-associated fraction) maintained a low baseline (< 1.9 mg L^-1^) during active growth, before increasing at 173 h during the late death phase (**Figure 2b**).

### 3.6 Nitrate leaching in soil columns

#### 3.6.1 Nitrate leaching dynamics for granular NP and granular N fertilizers in S1

The dynamics of NO_3_^-^ leaching in the S1 soil varied significantly depending on the fertilizer applied and the presence of stabilization treatments across five consecutive leaching episodes (**Figure 3**). The linear mixed-effects analysis confirmed significant effects of treatment, leaching event, and their interaction for both granular NP and granular N fertilizers (**Table 4**).

**Figure 3:**
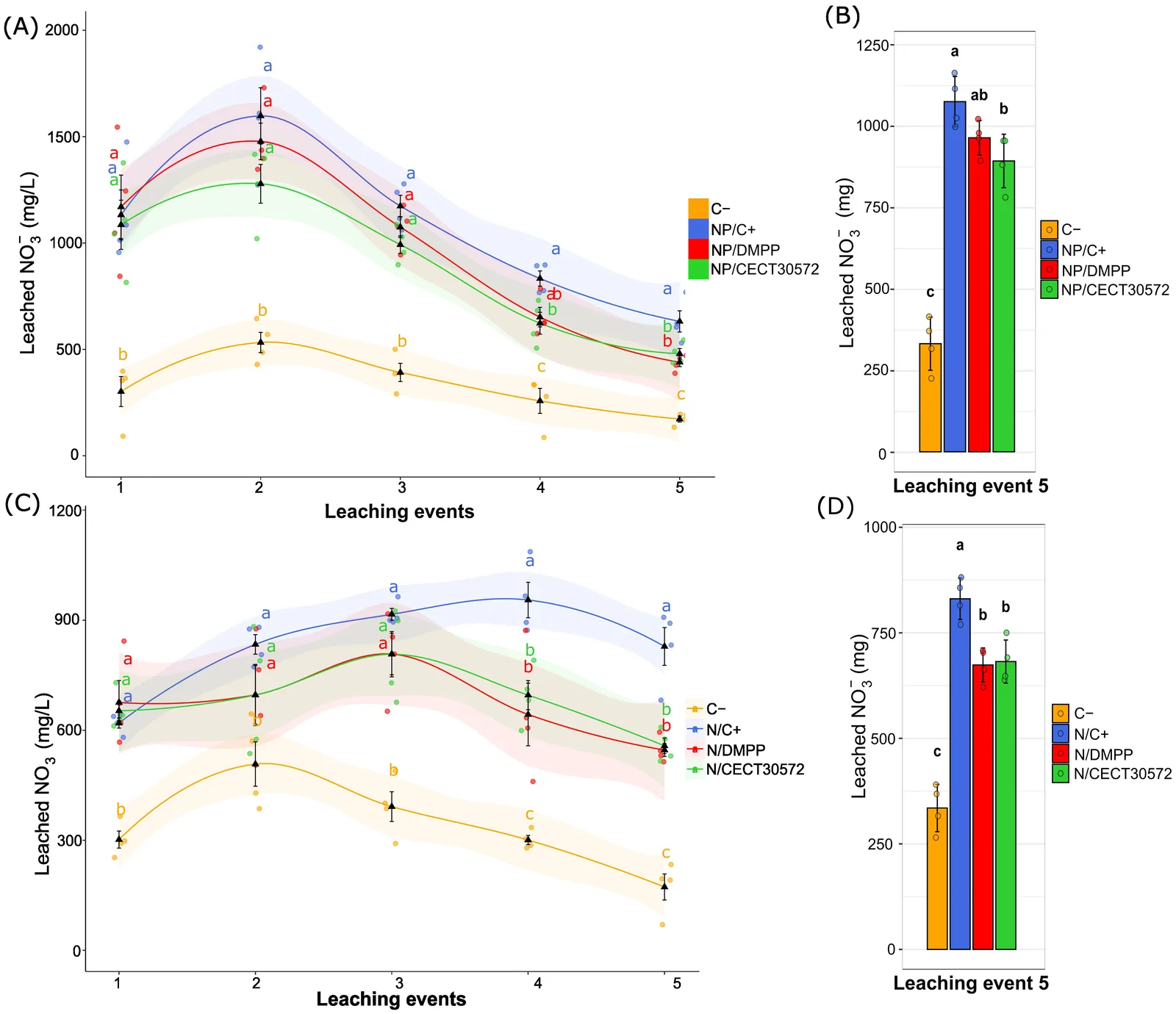
Nitrate concentration in leachates and cumulative mass of leached nitrate (**(A, B)** for treatments using granular NP fertilizer and **(C, D)** for treatments using granular N fertilizer) in columns containing substrate 1 (S1). Nitrate concentration was measured during the five leaching events, whereas cumulative nitrate mass represents the total mass leached at the end of the experiment (leaching event 5). Data are presented as mean ± SE (n = 4). Colored dots represent individual experimental replicates. Shaded areas indicate the 95% confidence interval for each treatment. Different letters indicate statistically significant differences among treatments, determined by the corresponding statistical analyses (p < 0.05).

**Table 4:** Linear mixed-effects model analysis of treatment, leaching event, and treatment × leaching event effects on nitrate (NO_3_^-^) concentrations for each soil–fertilizer combination.

Soil	Fertilizer	Effect	F	df1	df2	p-value
S1	NP	Treatment	45.715	3	13.611	<0.001
		Leaching event	64.788	4	40.339	<0.001
		Treatment × Leaching event	2.952	12	40.339	0.005
S1	N	Treatment	70.181	3	22.275	<0.001
		Leaching event	12.345	4	43.377	<0.001
		Treatment × Leaching event	2.841	12	43.377	0.006
S2	NP	Treatment	150.203	3	21.634	<0.001
		Leaching event	48.162	4	38.104	<0.001
		Treatment × Leaching event	5.419	12	38.104	<0.001
S2	UAN	Treatment	157.052	3	27.864	<0.001
		Leaching event	32.063	4	40.780	<0.001
		Treatment × Leaching event	4.843	12	40.780	<0.001

Treatment, leaching event, and their interaction were included as fixed effects, with soil column as the subject and an AR(1) covariance structure to account for repeated measurements. F-values and corresponding degrees of freedom (df) and p-values are shown. Significant effects are indicated by p < 0.05.

In the treatments fertilized with granular NP (NP) (**Figures 3a, b**), the concentration profiles in the leachate showed a parabolic trend, peaking in the second leaching before declining steadily until the fifth. The unfertilized control (C−) maintained the lowest NO_3_^-^ concentrations throughout the trial, never exceeding 532.7 mg L^-1^in any of the leaching events (**Figure 3a**). In contrast, all treatments supplemented with NP reached maximum concentrations in event 2, and the conventional application (NP/C+) showed the highest leaching peak in a single event, at 1598 mg L^-1^. Both stabilization strategies (CECT30572 and DMPP) succeeded in mitigating this concentration peak, reducing the values to levels of around 1278–1478 mg L^-1^. Follow-up comparisons performed for individual leaching events showed no statistically significant differences between the fertilized treatments during the first three events. In the fourth leaching event, the CECT30572 bio-coated formulation (NP/CECT30572) significantly reduced NO_3_^-^ leaching compared to the conventional control (p = 0.043), whereas the synthetic inhibitor (NP/DMPP) showed no statistically significant difference compared to NP/C+. In the fifth leaching event, both CECT30572 and DMPP significantly reduced NO_3_^-^ leaching concentrations compared with C+ (p = 0.019 and 0.004 respectively), while no significant difference was detected between CECT30572 and DMPP.

This trend was also reflected in the total accumulated mass of leached NO_3_^-^ (**Figure 3b**). The conventional NP/C+ treatment accumulated the greatest loss (1073.9 mg in event 5), whereas the CECT30572-coated fertilizer significantly reduced the total accumulated mass exported (887.6 mg) (p = 0.021). The DMPP treatment did not significantly differ from NP/C+ or NP/CECT30572 for this endpoint. Thus, CECT30572 showed a significant reduction in nitrate loss compared with the conventional control, while its response did not differ significantly from that observed with DMPP. When expressed relative to the total N applied, cumulative NO_3_^-^-N losses at the end of the experiment corresponded to 60.63% for NP/C+, 54.37% for NP/DMPP, and 50.35% for NP/CECT30572 (**Supplementary Table S3**).

Regarding the granular N-fertilized (N) treatments (**Figures 3c, d**), the leaching kinetics presented a more delayed, sustained progression. Instead of an immediate spike at event 2, NO_3_^-^ concentrations in the conventional N/C+ treatment continued to rise progressively, peaking at the fourth leaching event 955 mg L^-1^ (**Figure 3c**). Conversely, the two stabilized alternatives altered this kinetic profile, reaching their maximum values earlier (event 3, approx. 800 mg L^-1^) and entering a steady decline thereafter. During the final leaching stages, follow-up comparisons showed that both the CECT30572-coated and DMPP treatments had significantly lower NO_3_^-^ concentrations than the conventional formulation (p = 0.022 and p = 0.006, respectively, in the fourth leaching event; p < 0.001 for both treatments in the fifth event). No significant differences were detected between CECT30572 and DMPP in these events.

This temporary change was also reflected in the final cumulative loss profiles (**Figure 3d**). At the end of the experiment (leaching event 5), the conventional N/C+ treatment showed the highest cumulative nitrate loss (830.6 mg) whereas N/DMPP and N/CECT30572 showed lower cumulative losses of approximately 680 mg. Both stabilized treatments showed significantly lower cumulative nitrate losses than the conventional fertilizer (p = 0.005), while no significant difference was detected between N/CECT30572 and N/DMPP. When expressed relative to the total N applied, cumulative NO_3_^-^-N losses were 31.26% for N/C+, 25.36% for N/DMPP, and 25.66% for N/CECT30572 (**Supplementary Table S3**).

#### 3.6.2 Nitrate leaching dynamics for NP and UAN fertilizers in S2

The NO_3_^-^ leaching profiles recorded in the S2 soil showed temporal differences in nitrate losses among treatments with synthetic or biological stabilization (**Figure 4**). The linear mixed-effects analysis showed significant effects of treatment, leaching event, and their interaction for both NP and UAN fertilizers in S2 (**Table 4**). In general, the unfertilized control (C−) remained significantly below all nitrogen-fertilized groups throughout the five leaching events, with nitrate concentrations below 160 mg L^-1^ and a final cumulative nitrate loss of less than 100 mg (**Figure 4**).

**Figure 4:**
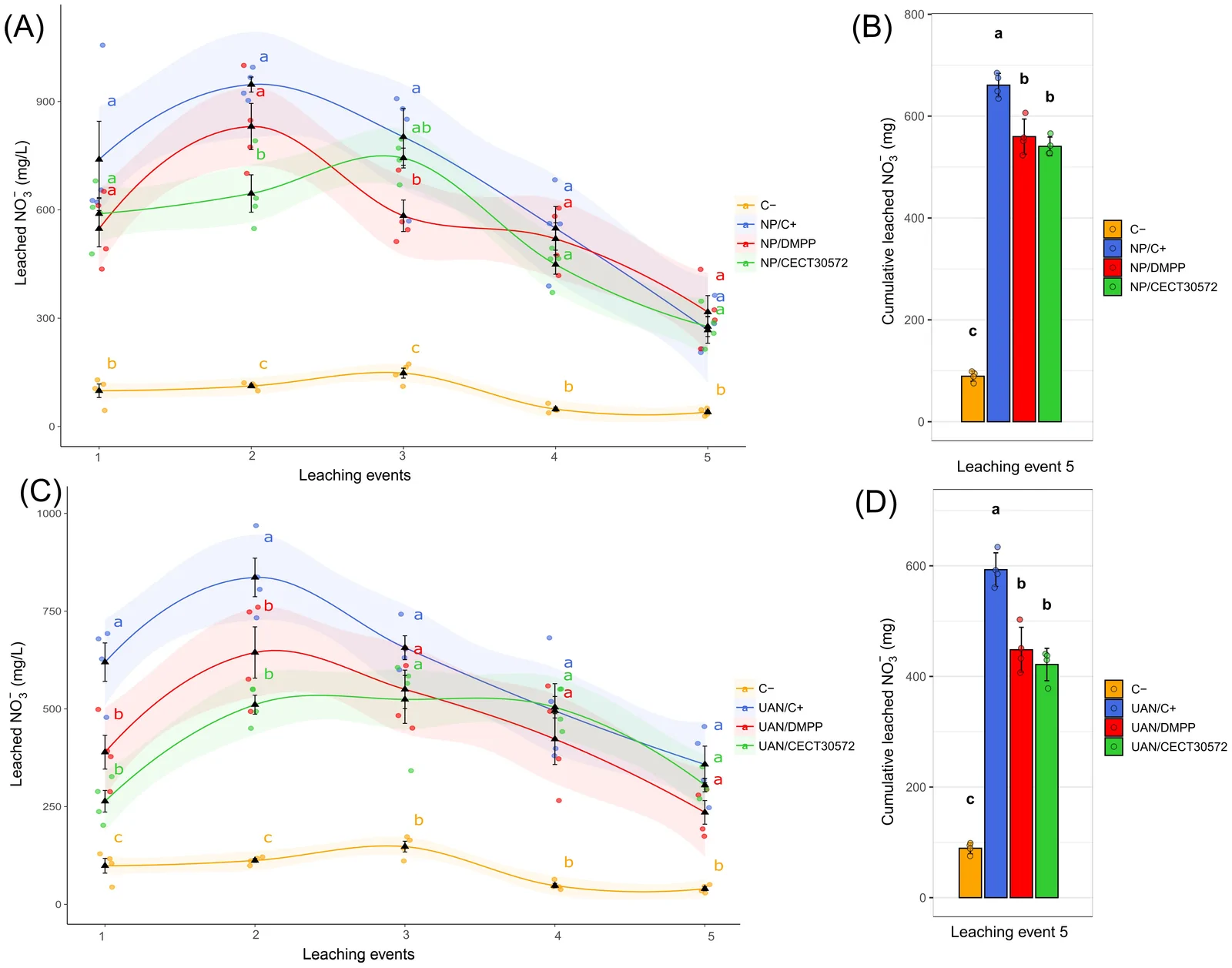
Nitrate concentration in leachates and cumulative mass of leached nitrate (**(A, B)** for treatments using granular NP fertilizer and **(C, D)** for treatments using liquid UAN fertilizer) in columns containing substrate 2 (S2). Nitrate concentration was measured during the five leaching events, whereas cumulative nitrate mass represents the total mass leached at the end of the experiment (leaching event 5). Data are presented as mean ± SE (n = 4). Colored dots represent individual experimental replicates. Shaded areas indicate the 95% confidence interval for each treatment. Different letters indicate statistically significant differences among treatments, determined by the corresponding statistical analyses (p < 0.05).

In the treatments fertilized with granular NP (NP) in S2 (**Figures 4a, b**), the nitrate concentration profiles showed differences in leaching dynamics among the stabilization treatments (**Figure 4a**). Follow-up comparisons for the first leaching event showed no statistically significant differences among the fertilized groups. In the second event, the bio-coated formulation (NP/CECT30572) significantly reduced NO_3_^-^ concentrations compared with the conventional fertilizer (NP/C+) (p = 0.01), whereas NP/DMPP did not differ significantly from NP/C+. Moreover, NP/CECT30572 showed significantly different NO_3_^-^ concentrations from NP/DMPP (p = 0.041). In the third leaching event, NP/DMPP significantly reduced NO_3_^-^ concentrations compared with NP/C+ (p=0.03), whereas NP/CECT30572 did not differ significantly from NP/C+; no significant difference was detected between NP/CECT30572 and NP/DMPP. In the fourth and fifth events, no statistically significant differences were detected among the fertilized treatments.

With regard to cumulative mass losses (**Figure 4b**), at the end of the experiment (leaching event 5), NP/C+ recorded the highest cumulative nitrate loss (660.8), whereas NP/DMPP and NP/CECT30572 showed lower cumulative losses of 559.75 and 540.65 mg, respectively. Both stabilization treatments showed significantly lower cumulative nitrate losses than the conventional fertilizer (p < 0.001), while no significant difference was detected between NP/DMPP and NP/CECT30572. When expressed relative to the total N applied, cumulative NO_3_^-^-N losses were 37.31% for NP/C+, 31.60% for NP/DMPP, and 30.52% for NP/CECT30572 (**Supplementary Table S3**).

As for the treatments fertilized with UAN in S2 (**Figures 4c, d**), the leaching dynamics revealed clear differences among treatments during the early stages of the trial. The conventional application (UAN/C+) showed a sharp peak in concentration at event 2, reaching 836.5 mg L^-1^. In this same event, the fertilizer mixed with CECT30572 (UAN/CECT30572) and that mixed with the chemical inhibitor (UAN/DMPP) reached concentrations of 510.7 and 644.2 mg L^-1^respectively, which were statistically lower than the conventional control (p<0.001), while no significant difference was detected between UAN/CECT30572 and UAN/DMPP. From the third to the fifth leaching events, follow-up comparisons showed no significant reduction in NO_3_^-^ leaching between either stabilization treatment and the conventional control.

With regard to cumulative nitrate losses (**Figure 4d**), at the end of the experiment (leaching event 5), UAN/C+ recorded a cumulative nitrate loss of 593 mg, whereas UAN/CECT30572 and UAN/DMPP showed lower cumulative losses of 421.6 and 448.3 mg, respectively. Both stabilization treatments showed significantly lower cumulative nitrate losses than the conventional fertilizer (p < 0.001), while no significant difference was detected between UAN/CECT30572 and UAN/DMPP. When expressed relative to the total N applied, cumulative NO_3_^-^-N losses were 20.92% for UAN/C+, 15.82% for UAN/DMPP, and 14.88% for UAN/CECT30572 (**Supplementary Table S3**).

### 3.7 Viable bacterial counts in fertilizer formulations during storage

The viable bacterial counts attributable to the CECT30572 inoculation in the different fertilizer formulations were determined at T0, 6 months, and 12 months of storage. At T0, the mean counts were 1.80×10^8^ CFU g^-^¹ in granular NP, 3.02×10^7^ CFU g^-^¹ in granular N, and 9.00×10^7^ CFU mL^-^¹ in UAN. After 6 months, the mean counts were 4.08×10^7^, 3.39×10^7^, and 6.05×10^6^ CFU g^-^¹ or CFU mL^-^¹ for granular NP, granular N, and UAN, respectively. At 12 months, the corresponding mean counts were 4.73×10^7^ CFU g^-^¹ for granular NP, 5.65×10^7^ CFU g^-^¹ for granular N, and 4.63×10^6^ CFU mL^-^¹ for UAN (**Table 5**). These values represent the mean of three independent fertilizer samples for each formulation and storage time, after correction for the corresponding non-inoculated fertilizer controls.

**Table 5:** Viable CECT30572 counts in fertilizer formulations during storage.

Fertilizer	T0		T 6 months		T 12 months	
	Mean	SD	Mean	SD	Mean	SD
Granular NP	1,80E+8	1,51E+07	4,08E+07	1,23E+06	4,73E+07	1,20E+06
Granular N	3,02E+07	3,63E+05	3,39E+07	2,56E+06	5,65E+07	7,45E+05
UAN	9,00E+07	8,57E+06	6,05E+06	3,21E+05	4,63E+06	6,56E+05

Mean viable counts of Bacillus subtilis CECT30572 and standard deviation (SD) in granular NP, granular N, and liquid UAN fertilizer formulations at T0, 6 months, and 12 months of storage. Granular fertilizer counts are expressed as CFU g^-^¹ and UAN counts as CFU mL^-^¹.

## 4 Discussion

The genomic architecture of *Bacillus subtilis* CECT30572 reveals a well-defined genetic repertoire associated with alternative nitrogen source utilization and metabolic homeostasis. A central finding in this study is the identification of a complete *nasABCDEF* gene cluster, which provides the molecular basis for the strain’s genetic potential for assimilatory nitrate reduction and, potentially, nitrate assimilation. In the genus *Bacillus*, the assimilatory reduction of nitrate (NO_3_^-^) is a two-step sequential process involving nitrate and nitrite reductases encoded by the *nas* pathway (Ogawa et al., 1995; Nakano et al., 1998). The presence of *nasBC* and *nasDE* is consistent with the presence of the catalytic components required for nitrate and NADH-dependent nitrite reduction, respectively, whereas *nasA* encodes a putative nitrate/nitrite transporter involved in the uptake of these substrates from the extracellular environment. Furthermore, the presence of *nasF* within this cluster is consistent with its proposed role in siroheme biosynthesis, a cofactor required for nitrite reductase activity (Ogawa et al., 1995; Nakano et al., 1998).

Interestingly, the divergent transcriptional orientation of the *nasA* gene relative to the rest of the other genes in the cluster is consistent with the genomic organization described in reference *B. subtilis* strains. The high sequence identity and structural collinearity of the *nas* region and its flanking regulatory genes between CECT30572 and the model strains 168 and NCIB 3610 indicate a high degree of conservation at the genomic level (Kunst et al., 1997; Zeigler et al., 2008). Protein sequence identities of 97–100% were observed for the six *nas* genes in these comparisons, further supporting the high similarity of the encoded proteins. These observations indicate conservation of the genetic potential associated with nitrogen metabolism within the compared *B. subtilis* strains. However, these genomic similarities do not demonstrate conservation of gene expression, enzymatic activity, or pathway function under specific environmental conditions (Earl et al., 2008; Mandic-Mulec et al., 2016).

The comparison with related taxa revealed greater differences in the organization of the nitrogen metabolism loci. In *Priestia megaterium*, the reduced syntenic conservation indicates greater divergence in the genomic organization of the compared region, although nitrogen metabolism loci, although components associated with nitrogen metabolism remain detectable (Eppinger et al., 2011; Gupta et al., 2020). This pattern is consistent with greater genomic divergence among more distantly related taxa, but does not by itself establish differences in pathway function or ecological adaptation. Similarly, no corresponding *nasBC* homologs were detected in the analyzed genomic region of *Bacillus amyloliquefaciens* DSM 7 under the alignment conditions used. This result indicates that homologs were not detected in the compared region, but cannot be interpreted as definitive evidence for their complete absence from the genome (Belbahri et al., 2017; Rückert et al., 2011). In CECT30572, the preservation of the *nasABCDEF* cluster and its associated regulatory genes indicates conservation of the genetic potential for nitrate assimilation and nitrogen regulation at the genomic level (Allard-Massicotte et al., 2016; Blake et al., 2021).

In addition to this core catalytic mechanism, the metabolic versatility of CECT30572 relies on its ability to adapt to changes in nutrient availability through a coordinated multi-level regulatory network. The identification of the global nitrogen regulator *tnrA*, and the key metabolic node CodY indicates that this strain possesses regulatory components associated with the hierarchical utilization of nitrogen sources (Sonenshein, 2007; He et al., 2023). Under nitrogen-limiting conditions, TnrA can activate alternative assimilation pathways, including the nas cluster, whilst repressing genes associated with the preferential utilization of nitrogen (Wray et al., 1996; Yoshida et al., 2003; Kayumov et al., 2011). Conversely, when intracellular nitrogen or energy levels are saturated, CodY integrates nutritional signals (GTP and branched-chain amino acids) to repress non-preferred pathways, promoting rapid growth and coordinating global metabolic adaptations (Sonenshein, 2007; Brinsmade et al., 2014; Barbieri et al., 2015; He et al., 2023). Although these regulatory functions are well established in *Bacillus*, their specific activity and regulatory interactions in CECT30572 were not experimentally assessed in this study.

This homeostatic balance is further reinforced by *glnR*, a *tnrA* homolog that acts as a transcriptional repressor of the *glnRA* glutamine synthetase operon and the *ureABC* urease cluster when preferred nitrogen sources are abundant, preventing futile metabolic cycles (Wray et al., 1997; Gunka and Commichau, 2012). Additionally, the genome harbors *gltC* (LysR family), which acts as the transcriptional activator of the *gltAB* glutamate synthase operon, potentially enabling downstream conversion of assimilated nitrogen into glutamate, the primary amino donor for cellular metabolism (He et al., 2023). Finally, the presence of nrgAB together with the AmtB ammonium transport system indicates the presence of genetic components associated with ammonium acquisition during nitrogen limitation (Detsch and Stülke, 2003; Kayumov et al., 2011). As for the other regulatory and transport genes, their presence in the genome does not demonstrate their expression or enzymatic activity under the experimental conditions investigated.

This complex genetic and regulatory system was further investigated through *in vitro* growth kinetics and measurements of nitrate and ammonium in CECT30572 cultures. The substantial reduction of approximately 455 mg L^-1^of NO_3_^-^ in the culture medium demonstrates nitrate depletion under the conditions tested and is compatible with the nitrate assimilation potential predicted from the presence of the *nasABCDEF* genes (Ogawa et al., 1995; Nakano et al., 1998). However, nitrate depletion alone does not demonstrate expression or catalytic activity of the complete *nasABCDEF* pathway. While NO_3_^-^ consumption occurred on the order of hundreds of milligrams per liter, the total accumulated intracellular-associated ammonium (NH_4_^+^) was much lower. During both exponential and stationary growth phases, no extracellular NH_4_^+^ was detected, and intracellular pools remained close to basal levels (< 1.9 mg L^-1^). Thus, the nitrate removed from the medium was not recovered predominantly as extracellular or intracellular-associated NH_4_^+^ under the conditions tested. Although its specific fate cannot be determined from the present measurements, we hypothesize that it could have been assimilated with the bacteria in the form of amino acids.

Spectrophotometric monitoring revealed that nitrite (NO_2_^-^) remained undetectable in the culture medium throughout the entire assay. Since genomic analyses demonstrated that CECT30572 possesses the genetic machinery for nitrate reduction to nitrite through both the assimilatory *nasBC* and respiratory *narGHJI* pathways, the physiological relevance of the *narGHJI* pathway under the aerobic conditions used in the present study remains uncertain. Respiratory nitrate reduction is generally associated with oxygen-limited conditions. Therefore, the presence of *narGHJI* should be interpreted as evidence of genetic potential rather than demonstrated respiratory nitrate reduction under the experimental conditions. The absence of detectable extracellular NO_2_^-^ therefore indicates that no measurable nitrite accumulated in the culture medium, but does not by itself establish the pathway responsible for nitrate reduction or exclude intracellular nitrite formation. Thus, the present data do not allow conclusions regarding intracellular nitrite dynamics or metabolic fluxes. Specifically, the coordinated activity of the glnRA and gltAB pathways could facilitate the assimilation of reduced nitrogen if the nitrate-derived nitrogen entered intracellular ammonium pools, thereby limiting the accumulation of free ammonium or nitrite intermediates within the cell (Detsch and Stülke, 2003; Commichau et al., 2008; Gunka and Commichau, 2012). This phenomenon is consistent with the high-affinity regulation of nitrogen assimilation characteristic of *Bacillus* species, in which intracellular NH_4_^+^ is tightly regulated to avoid toxicity (Fisher, 1999). However, the present data do not allow us to determine whether the nitrate-derived nitrogen was incorporated into cellular macromolecules or other intracellular N pools (Detsch and Stülke, 2003; Commichau et al., 2008; Gunka and Commichau, 2012).

The increase in intracellular-associated NH_4_^+^ observed at 173 h during the late death phase may reflect changes in nitrogen metabolism associated with prolonged starvation and declining cell viability. At this advanced stage of culture, active protein synthesis is expected to decrease, while intracellular macromolecule degradation and nitrogen recycling may contribute to the accumulation of free cytoplasmic NH_4_^+^ (Tam et al., 2007; Gunka and Commichau, 2012; Schultz et al., 2017). The coordinated activity of the *glnRA* and *gltAB* pathways may also contribute to the tight regulation and assimilation of intracellular reduced nitrogen under these conditions (Detsch and Stülke, 2003; Commichau et al., 2008). However, cell lysis, nitrogen recycling, and the potential release or accumulation of extracellular organic nitrogen compounds were not directly assessed in the present study. Therefore, these processes may have contributed to the observed late-stage nitrogen dynamics and cannot be excluded. Importantly, the relatively small intracellular-associated NH_4_^+^ pool compared with the total amount of NO_3_^-^ consumed indicates that it does not account for the nitrate removed during the assay. The fate of the remaining nitrate-derived nitrogen therefore remains unresolved.

From an environmental perspective, the genomic characteristics of CECT30572 strongly suggest that the strain lacks the canonical genetic machinery required for complete denitrification. In agricultural soils, native microbial communities are major drivers of nitrogen losses and greenhouse gas emissions through the sequential reduction of nitrate to gaseous nitrogen species, including nitric oxide (NO), nitrous oxide (N_2_O), and dinitrogen (N_2_) (Philippot et al., 2007; Butterbach-Bahl et al., 2013). In contrast, the genome of CECT30572 lacks the key genetic determinants required for these downstream denitrification steps, including the *nirK/nirS*, *norBC/norZ*, and *nosZ* gene clusters. Thus, the genomic evidence supports a low likelihood of canonical denitrification by CECT30572. Previous characterization of this strain by González-Andrés et al., 2025 reported no production of N_2_ from nitrate under the conditions tested and supported an assimilatory nitrate reduction to ammonium (ANRA) pathway. Moreover, that study reported reduced N_2_O emissions following the application of CECT30572 under soil–plant conditions, providing additional evidence that its application does not promote N_2_O emissions under the conditions evaluated. Nevertheless, the absence of annotated denitrification genes does not completely exclude alternative pathways or strain-specific genetic variants, and no direct measurements of NO, N_2_O, or N_2_ production by CECT30572 were performed in the present study. Therefore, the potential contribution of CECT30572 to gaseous nitrogen production cannot be conclusively excluded. Furthermore, although the genomic and previous experimental evidence supports the potential for nitrate assimilation and reduction to ammonium, the fate of the nitrate removed in the present study was not directly determined, and its incorporation into microbial biomass cannot be established from the present data.

From an agronomic perspective, the nitrate removal observed *in vitro* suggests that CECT30572 may contribute to reducing the availability of NO_3_^-^ for leaching (Cameron et al., 2013; G. P. Robertson and Vitousek, 2009). The substantial decrease in NO_3_^-^ concentration observed in the CECT30572 cultures, together with the lack of extracellular NO_2_^-^ accumulation, is compatible with nitrate assimilation by the strain under the controlled conditions of the *in vitro* assay. Nevertheless, the present experiments do not allow us to determine the proportion of removed nitrate that was incorporated into cellular biomass or other intracellular nitrogen pools.

Importantly, the *in vitro* findings were supported by soil column leaching experiments conducted under controlled soil-column conditions. Mineral fertilizer coated with CECT30572 significantly reduced NO_3_^-^ losses compared with the uncoated fertilizer treatment, with reductions that were in several cases not significantly different from those observed with the nitrification inhibitor 3,4-dimethylpyrazole phosphate (DMPP). These results highlight the potential of CECT30572 as a biological strategy for reducing nitrate leaching, with effects that were broadly similar to those observed with DMPP under the conditions tested. This similar effect on nitrate leaching should not be interpreted as evidence of a shared mechanism between CECT30572 and DMPP. Moreover, the two treatments were applied at their respective doses, expressed as DMPP amount and viable CECT30572 cells (CFU), respectively, and therefore their effects should not be interpreted as evidence of physicochemical dose equivalence. Therefore, the mechanisms underlying the observed differences in nitrate leaching cannot be directly compared. The nitrate depletion observed for CECT30572 under controlled *in vitro* conditions provides a plausible biological mechanism that could contribute to the measurable reduction in NO_3_^-^ leaching observed in the soil-column experiment. The growth kinetics observed *in vitro* cannot be directly extrapolated to the non-sterile soil-column system, where interactions with the native microbial community and the experimentally modified soil conditions may affect CECT30572 dynamics. In addition, competition with the native soil microbial community may limit the establishment, persistence, and activity of CECT30572 under field conditions. Although the indigenous microbial community was not characterized, all treatments were established using the same soil microbial community. Therefore, the reduction in NO_3_^-^ leaching observed following CECT30572 inoculation is a clear finding that may reflect either a direct effect of the inoculated strain or indirect effects mediated by changes in the indigenous microbial community. The present experimental design does not allow these contributions to be separated. Physicochemical retention processes, such as nitrate adsorption, may also contribute to the observed reduction in NO_3_^-^ leaching. Such changes seem to be triggered by the introduction of CECT30572. Thus, while the soil-column results demonstrate an effect of CECT30572 inoculation on nitrate leaching, the relative contribution of direct nitrate removal, indirect microbial effects, and physicochemical retention remains unresolved. In addition, ammonium retention, total mineral N, and total soil N were not quantified after incubation in the soil-column experiments. Therefore, although reduced NO_3_^-^ leaching indicates lower nitrate losses, the extent to which CECT30572 affected overall soil N conservation and the distribution of N among different soil N pools were not determined.

The storage stability of the inoculated fertilizer formulations further supports their potential use as a biological fertilizer stabilizer. Although viable counts attributable to the CECT30572 inoculation decreased during the first six months, counts remained relatively stable between 6 and 12 months, reaching approximately 10^7^ CFU g^-^¹ in the granular formulations and 10^6^ CFU mL^-^¹ in UAN after 12 months. This persistence is noteworthy given the potentially stressful physicochemical conditions of fertilizer matrices and is consistent with the recognized suitability of spore-forming *Bacillus* species survival in agricultural formulations. Similar viable populations in the order of 10^7^–10^9^ CFU g^-^¹ after 6–12 months have been reported for *Bacillus*-based formulations (Chatterjee et al., 2026). Thus, the initial decline followed by stabilization indicates that substantial viable bacterial populations associated with the CECT30572 inoculation were maintained during prolonged fertilizer storage. Because the viable counts were corrected against non-inoculated fertilizer controls, the observed populations were attributable to the inoculation above the background cultivable population; nevertheless, as strain-specific identification was not performed, these results are interpreted as viability associated with the CECT30572 inoculation rather than independently confirmed strain-specific counts. Importantly, they demonstrate viability during fertilizer storage but not strain-specific persistence or activity after application to soil, which was not assessed in the present study.

If nitrate -derived nitrogen was assimilated by CECT30572, this process could potentially represent a transient form of biological nitrogen retention rather than permanent nutrient sequestration. Microbial turnover and subsequent mineralization could return assimilated nitrogen to the soil mineral N pool, potentially contributing to the synchronization of nitrogen availability with crop demand (Kuzyakov and Xu, 2013; Schimel and Bennett, 2004). However, this interpretation remains hypothetical in the present study because microbial nitrogen immobilization and subsequent mineralization were not directly measured. Further experiments, ideally using isotopically labelled nitrogen, would be required to determine the fate of nitrate removed by CECT30572 and to quantify its incorporation into microbial biomass and subsequent release.

Crucially, previous plant-scale experiments with CECT30572 have provided complementary evidence suggesting a potential role in transient nitrogen retention without leading to nitrogen deprivation, while producing significant positive agronomic outcomes (González-Andrés et al., 2025). Microcosm experiments with maize demonstrated that CECT30572 significantly enhanced plant performance, with increases in fresh weight (11%), dry weight (17%), and plant height (20%) compared to conventional NPK fertilization. Furthermore, these assays documented a substantial increase in nitrogen use efficiency, with physiological efficiency (PE) rising by 42% and internal nitrogen utilization efficiency (IE) by 45% (González-Andrés et al., 2025). Field trials also reported yield increases of 5% (González-Andrés et al., 2025), providing complementary evidence of the agronomic potential of CECT30572. These results support its potential as a plant growth-promoting rhizobacterium (PGPR), although the mechanisms underlying these effects remain to be fully elucidated (Lv et al., 2025; Mazuecos-Aguilera et al., 2024; Ortiz-Liébana et al., 2022). These previously reported plant and field responses provide complementary evidence of the agronomic potential of CECT30572, whereas the present study specifically addresses nitrate retention and leaching under controlled soil-column conditions.

Notably, the NO_3_^-^-leaching mitigation effect exerted by CECT30572 was consistently observed across the different fertilizer formulations and in both soils evaluated, suggesting that its beneficial action is robust and not restricted to a specific nitrogen source. However, differences were evident in the temporal dynamics of this response. In Soil 1, both CECT30572 and DMPP predominantly reduced NO_3_^-^ losses during the later leaching events, whereas in Soil 2 the effect became apparent during the earliest events. The inclusion of granular NP in both soils provides a common fertilizer treatment for assessing the influence of soil conditions on nitrate leaching and stabilization performance. However, because granular N and UAN were evaluated in S1 and S2, respectively, their responses cannot be directly attributed to soil type alone. For the common NP treatment, the markedly lower NO_3_^-^ leaching observed in Soil 2 compared with Soil 1 indicates that soil properties substantially influenced overall N dynamics, independently of the stabilization treatment. Despite these differences in absolute nitrate losses, the relative reductions associated with CECT30572 and DMPP compared with the corresponding control were broadly maintained across the two soils, supporting the consistency of the stabilization effect under different soil conditions. Differences in soil texture, particularly the higher clay content of Soil 2, could influence water flow and solute transport through effects on hydraulic conductivity and pore structure (Feng et al., 2024), potentially contributing to the lower overall leaching observed in Soil 2. However, these interpretations should be considered in the context of the experimental system, as soil preparation and column packing may have altered soil structure and water flow relative to intact agricultural soil. Differences in organic matter content and nitrogen status may represent additional factors contributing to the contrasting temporal responses observed between soils. Soil 1 exhibited substantially higher organic matter content, total nitrogen concentration, and C/N ratio than Soil 2, conditions generally associated with greater microbial biomass and enhanced microbial nitrogen immobilization capacity (Wang and Song, 2024). Under these conditions, the buffering effect of the native soil microbial community could contribute to a delayed manifestation of treatment differences until later stages of the leaching process, as microbial biomass can act as a temporary sink for mineral nitrogen through rapid immobilization processes (Beeckman et al., 2024; Wang and Song, 2024). Conversely, the lower organic matter and nitrogen contents of Soil 2 may be associated with a lower capacity for microbial and physicochemical buffering of mineral N, potentially contributing to the earlier emergence of treatment effects (Koch and Sessitsch, 2024; Wang and Song, 2024). However, this interpretation remains tentative because only two soils were evaluated and several soil properties differed simultaneously, including texture, micronutrient concentrations, electrical conductivity, and exchangeable cations. Therefore, the observed temporal differences cannot be attributed specifically to organic matter or nitrogen status. Although differences in soil texture, particularly the higher clay content of Soil 2, could also influence water flow and solute transport through effects on hydraulic conductivity and pore structure (Hillel, 2003; Jury and Horton, 2004), the contribution of the other differing soil properties cannot be excluded.

Finally, although cumulative NO_3_^-^ leaching at the end of the experiment indicated similar overall performance of CECT30572 and DMPP, with both treatments consistently reducing nitrate losses relative to the corresponding untreated fertilizer control, a clear tendency for earlier action of CECT30572 was observed, particularly in Soil 2 under NP fertilization, where statistically significant differences were detected during the initial leaching events. This early-stage mitigation effect may be relevant from an agronomic perspective, since the period immediately following fertilization is typically characterized by low crop nitrogen uptake capacity, when the risk of NO_3_^-^ losses is highest (Ju et al., 2009; Cui et al., 2018). During later growth stages plant demand for nitrogen increases, potentially reducing the relative necessity for strong nitrification or leaching control, and thereby indicating a potential relevance of early-stage NO_3_^-^ conservation (Subbarao et al., 2012). However, whether this temporal pattern provides a consistent agronomic advantage would require confirmation under a broader range of soil conditions and field environments.

Overall, the combined genomic, physiological, and soil-scale evidence indicates that *Bacillus subtilis* CECT30572 has the genetic potential for nitrate assimilation, directly demonstrates nitrate depletion under the *in vitro* conditions tested, and shows reduced nitrate leaching in soil columns. The observed nitrate depletion may contribute to the reduced nitrate losses in soil, although the relative contribution of biological nitrate removal, indirect microbial effects, and physicochemical retention processes remains unresolved.

## 5 Conclusions

This study indicates the potential of *Bacillus subtilis* CECT30572 as a bio-based fertilizer stabilizer capable of reducing nitrate losses from agricultural systems. Hybrid whole-genome sequencing revealed the genetic potential for nitrate assimilation, including the *nasABCDEF* operon and its associated regulatory network, while confirming the absence of the canonical genes required for complete denitrification.

The genomic predictions were supported by *in vitro* assays, which showed substantial nitrate depletion without detectable accumulation of extracellular nitrite or ammonium, a result compatible with nitrate assimilation by CECT30572 but not sufficient to demonstrate this process directly.

In the soil column leaching experiment, fertilizer coating or mixing with CECT30572 reduced cumulative nitrate leaching across contrasting soil types and fertilizer formulations, with reductions of a magnitude similar to those observed with the synthetic nitrification inhibitor 3,4-dimethylpyrazole phosphate (DMPP) under several experimental conditions. Overall, the results indicate that the nitrate depletion observed under *in vitro* conditions may contribute to the reduced nitrate leaching observed in soil columns. However, the fate of the depleted nitrate and the specific mechanisms underlying the reduction in nitrate leaching were not directly assessed, and other biological or physicochemical processes may also contribute to the observed response. Therefore, CECT30572 represents a promising biological approach for improving nitrogen retention and potentially reducing nitrate losses, although further studies are needed to elucidate the mechanisms involved.

## Data Availability

The datasets presented in this study can be found in online repositories. The names of the repository/repositories and accession number(s) can be found in the article/**Supplementary Material**.
