# Supplementary material for: Bacillus subtilis CECT30572 for biological fertilizer stabilization: an alternative to synthetic nitrification inhibitors
Source: Front Plant Sci. 2026 Oct 29;17:1936312. doi: 10.3389/fpls.2026.1936312 (PMC13754897; doi:10.3389/fpls.2026.1936312)
Supplement: Supplementary file 1 [file Table1.docx]

Supplementary Material

**Supplementary Table S1.** Genome-based taxonomic comparison of Bacillus subtilis CECT30572 with closely related reference genomes.

| **Reference genome** | **ANI (%)** | **dDDH d0 (%)** | **dDDH d4 (%)** | **dDDH d6 (%)** | **G+C difference (%)** |
| --- | --- | --- | --- | --- | --- |
| *B. subtilis* ATCC 6051 | 98.67 | 94.1 | 90.0 | 95.5 | 0.32 |

ANI, average nucleotide identity; dDDH, digital DNA–DNA hybridization; G+C, guanine plus cytosine. ANI was calculated using FastANI using the CECT30572 and B. subtilis ATCC 6051 genomes. dDDH values were obtained using the TYGS platform and are reported according to the three GBDP formulas (d0, d4, and d6).

**Supplementary Figure S1. Whole-genome sequence-based phylogenomic placement of** Bacillus subtilis **CECT30572.** Phylogenomic tree generated using the Genome BLAST Distance Phylogeny (GBDP) approach implemented in the Type (Strain) Genome Server (TYGS), based on whole-genome sequences. Bacillus subtilis CECT30572 is shown together with closely related reference genomes. Branch support values are indicated at the corresponding nodes.

**Supplementary Table S2.** Protein sequence identity of genes involved in assimilatory nitrogen metabolism among representative Bacillus genomes.

| **Gene** | **B. subtilis 168** | **B. subtilis NCIB 3610** | **B. subtilis subsp. spizizenii** | **B. velezensis** | **B. amyloliquefaciens** | **B. cabrialesii** | **P. megaterium** |
| --- | --- | --- | --- | --- | --- | --- | --- |
| **nasF** | 100% | 100% | 96% | 72% | 72% | 96% | — |
| **nasE** | 99% | 99% | 98% | — | — | 95% | — |
| **nasD** | 100% | 100% | 96% | — | — | 95% | — |
| **nasC** | 98% | 98% | 90% | — | — | 90% | — |
| **nasB** | 97% | 97% | 72% | 86% | 85% | 96% | — |
| **nasA** | 97% | 97% | 92% | 74% | 74% | 90% | 30% |

Protein sequence identity values were obtained from pairwise protein alignments performed using clinker. Values represent the percentage of identical amino acids between the CECT30572 protein and the corresponding homologous protein identified in each reference locus. A dash indicates that no alignment was detected by clinker above the selected alignment threshold.

**Supplementary Table S3.** Cumulative nitrate-N losses expressed as a percentage of applied nitrogen in the soil-column experiments.

| **Treatment** | **Soil1** | | **Soil2** | |
| --- | --- | --- | --- | --- |
|  | **NP fertilizer** | **N fertilizer** | **NP fertilizer** | **UAN fertilizer** |
| **C-** | N/A | N/A | N/A | N/A |
| **C+** | 60,62 | 31,26 | 37,30 | 20,92 |
| **DMPP** | 54,36 | 25,35 | 31,59 | 15,81 |
| **CECT30572** | 50,35 | 25,66 | 30,52 | 14,87 |

Values represent cumulative NO₃⁻-N losses at the final leaching event, expressed as a percentage of the total nitrogen applied with the corresponding fertilizer treatment: (cumulative NO3−−N leached/applied N)×100(cumulative\ NO₃⁻-N\ leached / applied\ N) × 100. NO₃⁻ concentrations were converted to NO₃⁻-N using the molecular mass ratio of N to NO₃⁻ (14/62). The C− treatment did not receive fertilizer and was therefore not expressed as a percentage of applied N. Values are presented without replacing the absolute nitrate-loss analysis reported in the main manuscript.
